# Methane Cracking over Cobalt Molybdenum Carbides

**DOI:** 10.1007/s10562-018-2378-4

**Published:** 2018-04-21

**Authors:** I. Alshibane, S. Laassiri, J. L. Rico, J. S. J. Hargreaves

**Affiliations:** 1 WestCHEM, School of Chemistry, University of Glasgow, Joseph Black Building, Glasgow, G12 8QQ UK; 2 Laboratorio de Catálisis, Facultad de Ingeniería Química, Universidad Michoacana de San Nicolás de Hidalgo, Edif. V1, C.U., 58060 Morelia, Michoacán Mexico

**Keywords:** Methane cracking, Catalytic decomposition, Cobalt molybdenum carbide, Cobalt molybdenum nitride

## Abstract

**Abstract:**

The catalytic behaviour of Co_3_Mo_3_C, Co_6_Mo_6_C, Co_3_Mo_3_N and Co_6_Mo_6_N for methane cracking has been studied to determine the relationship between the methane cracking activity and the chemical composition. The characterisation of post-reaction samples showed a complex phase composition with the presence of Co_3_Mo_3_C, α-Co and β-Mo_2_C as catalytic phases and the deposition of different forms of carbon during reaction.

**Graphical Abstract:**

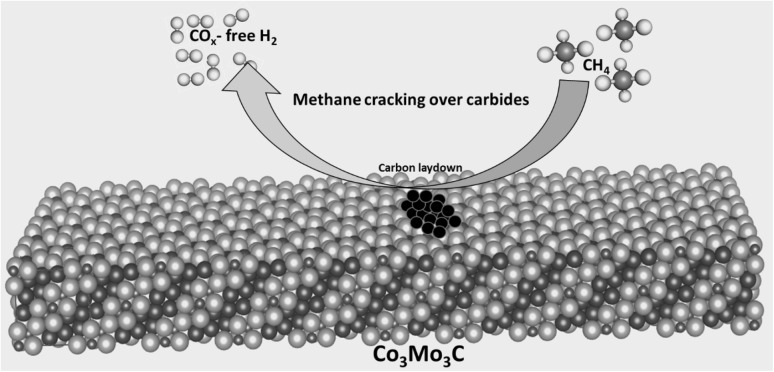

**Electronic supplementary material:**

The online version of this article (10.1007/s10562-018-2378-4) contains supplementary material, which is available to authorized users.

## Introduction

The design of novel and efficient materials for catalytic reactions is of major interest. Although, many of the materials studied in heterogeneous catalysis are metal and metal oxide based, attention has been directed towards the development of entirely novel catalyst families that could display modified performance. Examples of the materials investigated include carbides, nitrides and boron alloys [1–3]. Amongst these materials, carbides have arguably received the most attention due to the perceived analogies between their behaviour and that of precious metals, suggesting them to be potential replacements. In this context, the presence of interstitial carbon species has been argued to modify the electronic properties of the parent metal in systems such as those based upon molybdenum or tungsten, making them akin to precious metals such as platinum [4, 5].

Within the literature, metal carbides are known to possess activity for a wide range of applications including Fischer–Tropsch processes [6–8], hydrogenation [9–11], dehydroaromatisation [12], oxygen and hydrogen evolution [13] and ammonia decomposition [14, 15]. In more recent work, the reactivity of cobalt molybdenum carbide was studied for ammonia synthesis to gain an enhanced understanding of the potential role of lattice composition (i.e. the influence of the presence of interstitial carbon versus interstitial nitrogen) upon catalytic activity [16]. Although Co_3_Mo_3_C was found to be active for ammonia synthesis, the material was only active at higher temperature (500 °C) compared to its nitride counterpart which is active at 400 °C, and such activity was associated with the presence of some lattice nitrogen residing in 16c Wyckoff sites as determined from in-situ neutron diffraction studies.

In the current manuscript, comparison is made between the performance of Co_3_Mo_3_C and Co_6_Mo_6_C for methane cracking to determine the role of stoichiometry and such performance is contrasted with their isostructural nitride counterparts. The methane cracking reaction is of potential interest as an environmentally friendly approach for CO_x_-free hydrogen production [17–19].

## Experimental

### Materials Preparation

The preparation of cobalt molybdenum related materials has been documented in detail in our previous work [16, 20]. In a typical synthesis, CoMoO_4_ was prepared by adding 5.59 g of Co(NO_3_)_2_·6H_2_O (> 98%, Sigma-Aldrich) and 4.00 g of (NH_4_)_6_Mo_7_O_24_·4H_2_O (99.98%, Sigma-Aldrich) dissolved in 200 mL of deionized water. The solution was then heated to 85 °C and held at this temperature for 5 h. The resulting purple precipitate was filtered and washed with deionized water and ethanol. The precipitate was then calcined in static air at 500 °C for 5 h to obtain dehydrated cobalt molybdate. Co_3_Mo_3_N was prepared by ammonolysis of CoMoO_4_ under NH_3_ (BOC, 99.98%) at a flow rate of 100 mL min^−1^ at 785 °C for 5 h. The temperature was increased from ambient to 357 °C at 5.6 °C min^−1^, then after to 447 °C min^−1^ at 0.2 °C min^−1^ before being finally increased to 785 °C at 2.1 °C min^−1^. Co_3_Mo_3_C was prepared by the carburization of Co_3_Mo_3_N under 20 vol% CH_4_ in H_2_ (BOC, 99.98%) at a flow rate of 12 mL min^−1^ at 700 °C for 2 h with a ramp rate of 6 °C min^−1^ to reach 350 °C followed by 1 °C min^−1^ to attain 700 °C. Co_6_Mo_6_C was prepared by reducing Co_3_Mo_3_C at 900 °C for 5 h under 60 mL min^−1^ of a 75 vol% H_2_ in Ar (BOC, 99.98%) gas mixture.

### Materials Characterization

X-ray diffraction patterns were collected on a Panalytical X’Pert PRO instrument, using Cu Kα radiation (λ = 0.154 nm) over a 2θ range of 5°–85°, a step size of 0.0167°, and a counting time of 1 s per step. Samples were prepared by compaction into a Si sample holder. Phase identification was obtained by comparison with JCPDS database files. The surface areas of the samples were determined by application of the BET method to N_2_ physisorption isotherms collected at − 196 °C upon samples previously degassed at 110 °C under vacuum for 12 h. Scanning electron microscopy was performed on Philips XLSEM and FEI Quanta 200F Environmental instruments operating at 20 kV for the investigation of morphology. Samples were coated with an Au/Pd alloy prior to imaging. CHN analysis was undertaken by combustion using an Exeter Analytical CE-440 Elemental Analyzer. Thermogravimetric analysis (TGA) was carried out using a TA instruments QA instrument with measurements being undertaken in temperature range from room temperature to 1000 °C (ramp rate 10 degrees per minute) under a flow rate of 50 mL min^−1^ of air. Raman spectra of the samples were recorded at room temperature on a Horiba Jobin Yvon LabRam HR confocal Raman microscope, using a laser excitation of 523 nm.

### Catalytic Activity Tests

Methane cracking reactions were performed using 0.2 g of material which was placed in a quartz tube reactor under a gas feed of 12 mL min^−1^ of a mixture of 75 vol% CH_4_ in N_2_ (BOC, 99.98%) at 800 °C for 8 h on stream. Hydrogen production was monitored by online gas chromatography (GC) using a TCD with Ar as carrier gas and employing a Molecular Sieve 13× column. The gas exhaust was also analysed in a periodic manner for the determination of CO_*x*_ by off-line FTIR analyses employing a gas-phase FTIR cell which could be isolated and by-passed for off-line analyses. FTIR spectra were recorded using a FTIR-8400S, Shimadzu apparatus. Each spectrum was collected at a spectral resolution of 2 cm^−1^, applying a scan range of 500–3500 cm^−1^.

## Results

The structural and textural properties of the cobalt molybdenum based materials following the different nitridation and carburisation processes were monitored using a range of techniques. The results of the structural characterisation by XRD have been reported previously [16]. Powder XRD and neutron diffraction studies have confirmed the formation of high quality pure phases of Co_3_Mo_3_C, Co_6_Mo_6_C, Co_3_Mo_3_N and Co_6_Mo_6_N. These results can be found in the Supplementary Information (Figs. S1, S2).

**Fig. 1:**
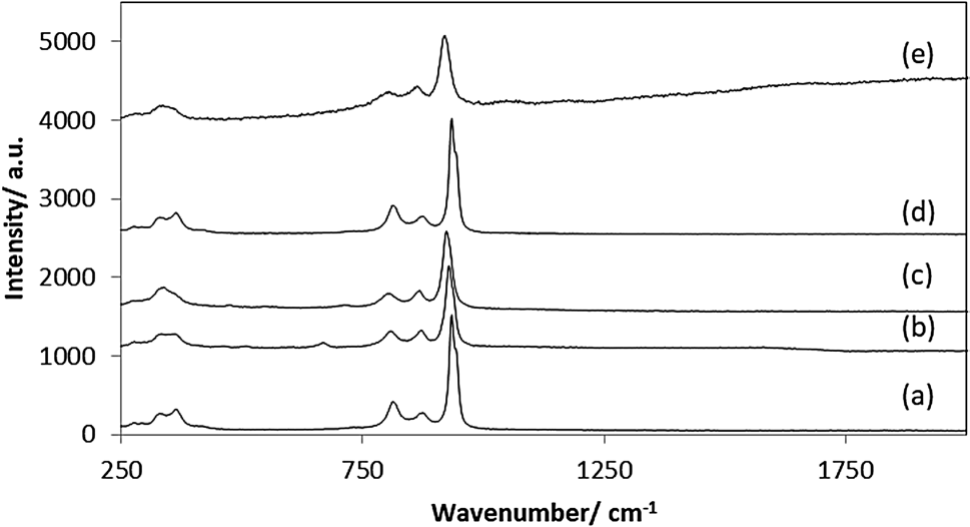
Raman spectra of as prepared materials (*a*) CoMoO_4_, (*b*) Co_3_Mo_3_N, (*c*) Co_3_Mo_3_C, (*d*) Co_6_Mo_6_N and (*e*) Co_6_Mo_6_C

Raman spectra of the pre-reaction cobalt molybdenum materials are presented in Fig. 1. The Raman bands at 354, 806, 866, 930 and 941 cm^−1^ are consistent with the presence of a surface cobalt molybdate phase [21] as might be expected resulting from the presence of a surface passivation layer [22] for the carbide and nitride materials, which are known to be air-sensitive. Importantly, no Raman bands related to carbon deposition during the synthesis of Co_3_Mo_3_C and Co_6_Mo_6_C samples were observed which is consistent with the preparation of non-coked materials.

**Fig. 2:**
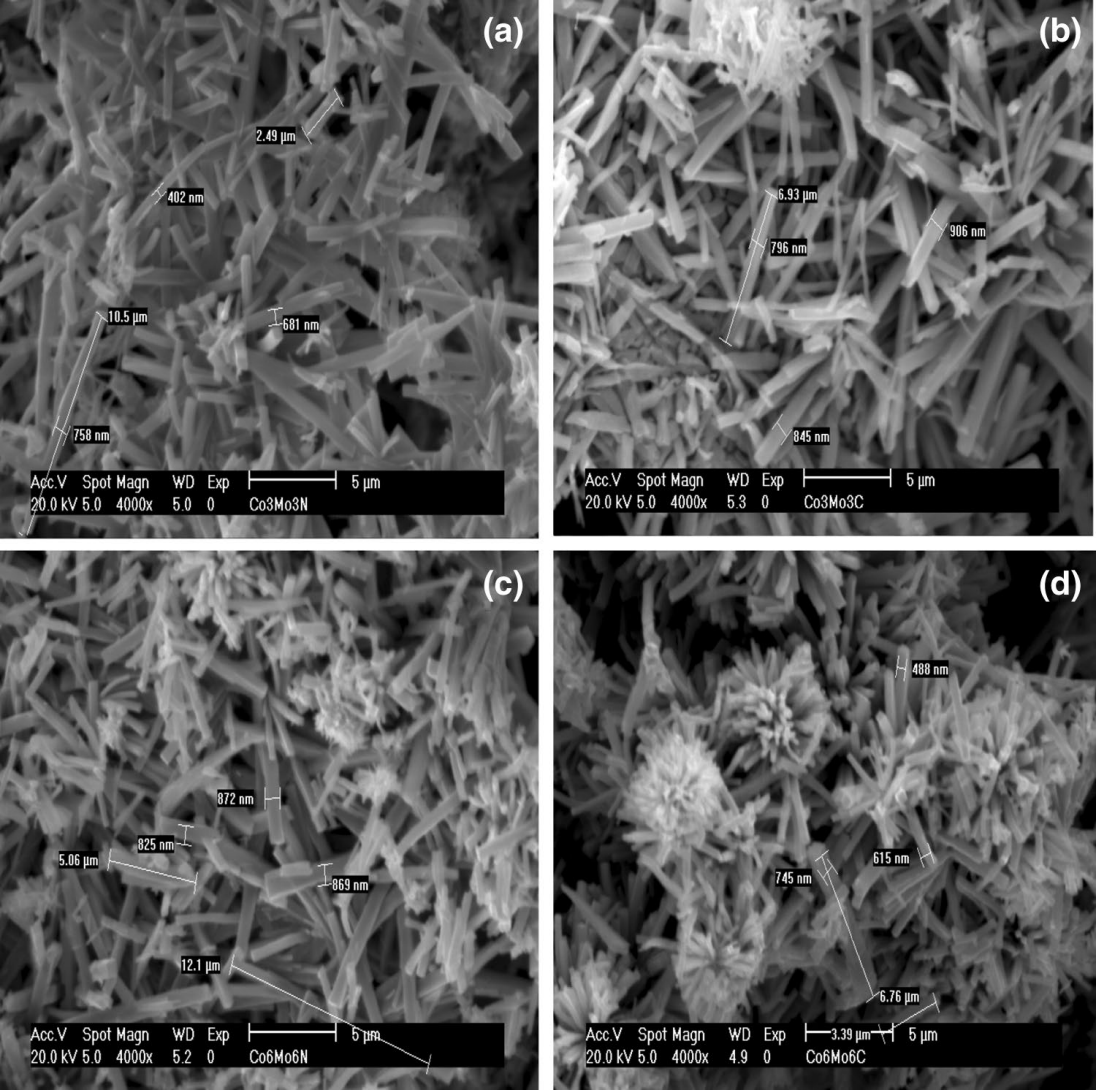
SEM images of as prepared materials: **a** Co_3_Mo_3_N, **b** Co_3_Mo_3_C, **c** Co_6_Mo_6_N and **d** Co_6_Mo_6_C

The SEM images presented in Fig. 2 demonstrate that all the materials prepared are pseudomorphic and exhibit a needle-like morphology as reported elsewhere [16]. The surface areas of the materials were determined to be 7, 18, 4 and 13 m^2^ g^−1^ for CoMoO_4_, Co_3_Mo_3_N, Co_6_Mo_6_N and Co_3_Mo_3_C respectively (Table 1). The lower surface area of the Co_6_Mo_6_C phase (∼ 3 m^2^ g^−1^), can be attributed to the higher reaction temperature applied in its preparation.

**Table 1:** Summary of the textural and structural characterisation of post-reaction catalysts

	Post-reaction XRD phase	Nitrogen content^a^/wt%		Carbon content^a^/wt%		S_BET_^b^/m^2^ g^−1^	
		As-prepared	Post-reaction	As-prepared	Post-reaction	As prepared	Post-reaction
CoMoO_4_	Graphite (003-0401), β-Mo_2_C (001-1188), α-Co (01-089-7093)	–	N.d.	–	71 ± 1	7	31
Co_3_Mo_3_N	Co_3_Mo_3_C (03-065-7128), graphite (003-0401), β-Mo_2_C (001-1188), α-Co (01-089-7093)	3.0 ± 0.1 N	N.d.	–	85 ± 3	18	63
Co_3_Mo_3_C	Co_3_Mo_3_C (03-065-7128), graphite (003-0401), β-Mo_2_C (001-1188), α-Co (01-089-7093)	–	N.d.	2.5 ± 0.1	84 ± 1	13	50
Co_6_Mo_6_N	Co_3_Mo_3_C (03-065-7128), graphite (003-0401), β-Mo_2_C (001-1188), α-Co (01-089-7093)	1.6 ± 0.1 N	N.d.	–	84 ± 1	4	59
Co_6_Mo_6_C	Co_3_Mo_3_C (03-065-7128), graphite (003-0401)	–	N.d.	1.3 ± 0.1	69 ± 1	3	24

*N.d*. not detected

^a^Nitrogen analysis undertaken using an Exeter Analytical CE-440 Elemental Analyser

^b^S_BET_ is the specific surface area evaluated using the BET model

**Fig. 3:**
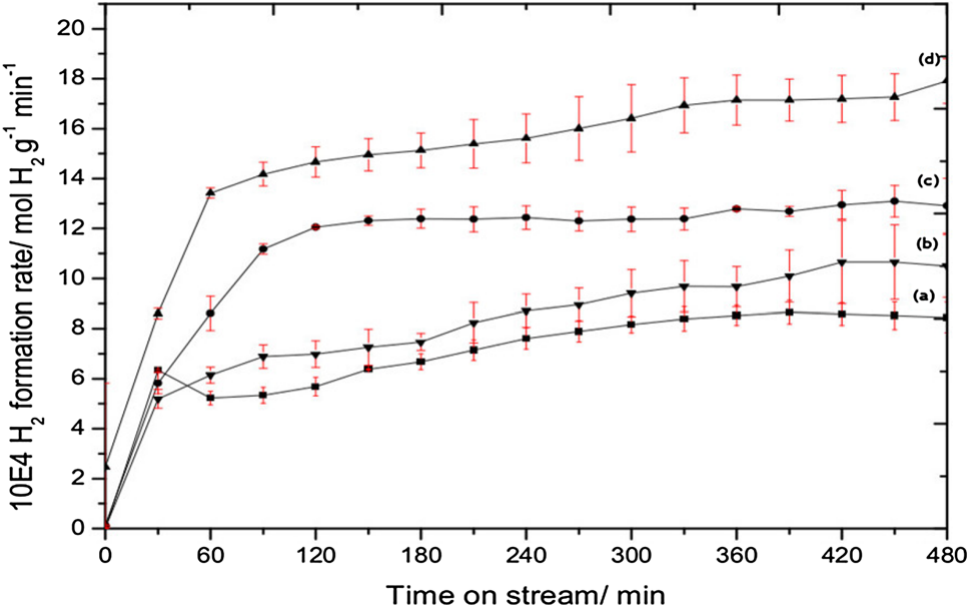
Hydrogen formation rates as a function of time on stream for methane cracking over (*a*) CoMoO_4_, (*b*) Co_6_Mo_6_C, (*c*) Co_3_Mo_3_N and (*d*) Co_6_Mo_6_N at 800 °C

The role of the nature of the stoichiometry and also the interstitial element present in the catalytic methane cracking activity of cobalt molybdenum materials was investigated by comparing the activity of the Co_3_Mo_3_C and Co_6_Mo_6_C to Co_3_Mo_3_N and Co_6_Mo_6_N materials. The reaction profiles illustrating the evolution of the mass normalised hydrogen formation rate as a function of time on stream is presented Fig. 3. Although, all materials displayed activity for hydrogen production, clear differences in the hydrogen formation rates are observed. Hydrogen production tended to reach a plateau after 120 min on stream. However, in the case of the Co_3_Mo_3_C sample, less reproducible behaviour occurred, which was possibly related to carbon build-up during the reaction resulting in reactor blockage. Similar observations were witnessed upon repeating the experiment and the structural and textural properties of the post-reaction samples were all similar (Fig. S3). Due to the fact that the performance of Co_3_Mo_3_C is less reproducible, its reaction profile is not presented in Fig. 3 but is instead shown in Fig. S2.

In the presence of N_2_ and H_2_, as is the case in this study, Co_3_Mo_3_N and Co_3_Mo_3_C have been reported to be active catalytic systems for ammonia generation at ambient pressure [16]. However, the reaction is thermodynamically unfavourable at high temperature and ammonia content at equilibrium is ~ 0.001 mol% at ca. 800 °C. Thus, the consumption of hydrogen to generate ammonia by reaction with the N_2_ internal standard within the methane feed under these conditions can be safely ruled out.

**Fig. 4:**
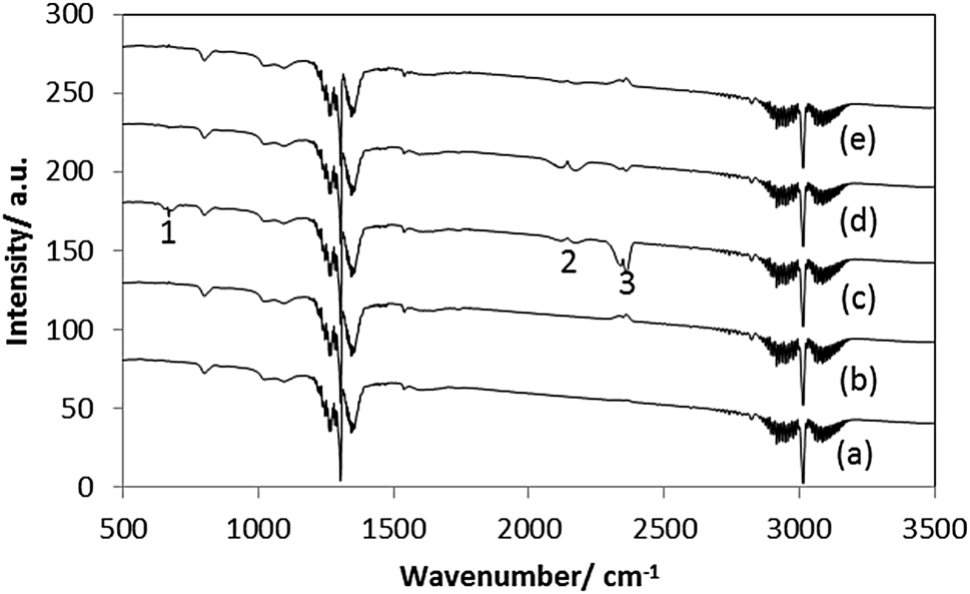
FTIR analyses of gas-phase products from CoMoO_4_ reacted with CH_4_/N_2_ (*a*) the feed gas, (*b*) 800 °C, (*c*) 800 °C 20 min, (*d*) 800 °C 50 min, and (*e*) 800 °C 60 min. Bands 1 and 3 are related to gas-phase CO_2_ whereas band is 2 related to gas-phase CO

In the case of cobalt molybdenum nitride and carbides, off-line FTIR spectra recorded periodically during reaction showed that the production of CO and CO_2_ during methane cracking was below the detection limit. However, as might be expected, CO and CO_2_ were clearly observed when CoMoO_4_ was used as a catalyst (Fig. 4). FTIR analysis of gas products from this sample shows after 20 min of reaction, bands at 660 and 2360 cm^−1^ which can be related to CO_2_ and at 2177 cm^−1^ which is assigned to CO. However, the production of CO and CO_2_ ceased after 50 min of reaction. It is noteworthy that the production of CO, even in small concentrations, can be harmful in the case of some downstream applications such as the use of H_2_ in PEM fuel cells [23, 24]. The results presented herein suggest, beyond the initial loss of the surface passivation layer upon reaction, the use of cobalt molybdenum nitrides or carbides might be suitable for the production of CO_x_-free hydrogen from methane cracking for such applications.

**Fig. 5:**
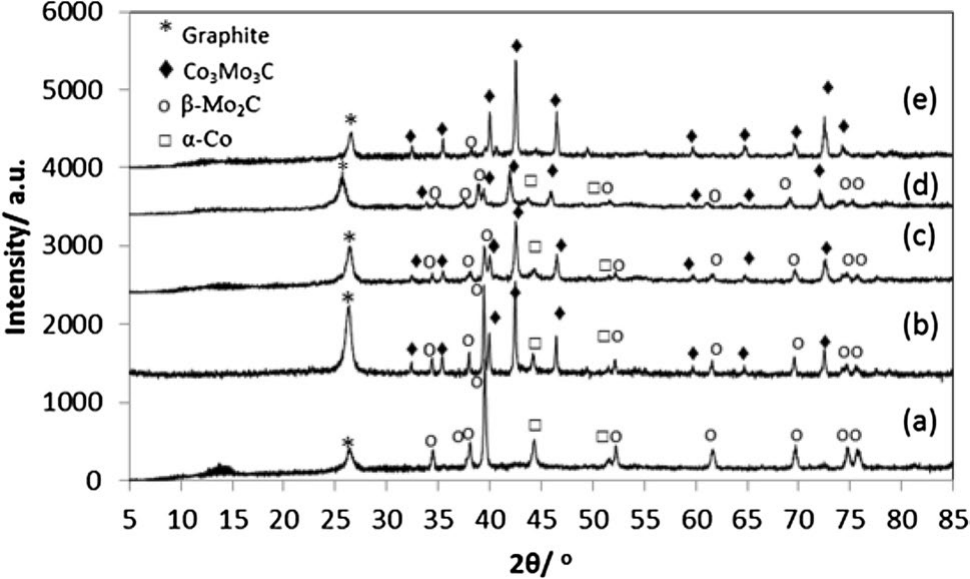
Powder X-ray diffraction patterns of the post-reaction: (*a*) CoMoO_4_, (*b*) Co_3_Mo_3_N, (*c*) Co_3_Mo_3_C, (*d*) Co_6_Mo_6_N and (*e*) Co_6_Mo_6_C materials. Diamond: Co_3_Mo_3_C (03-065-7128), asterisk: graphite (003-0401), open circle: β-Mo_2_C (001-1188), open square: α-Co (01-089-7093)

**Fig. 6:**
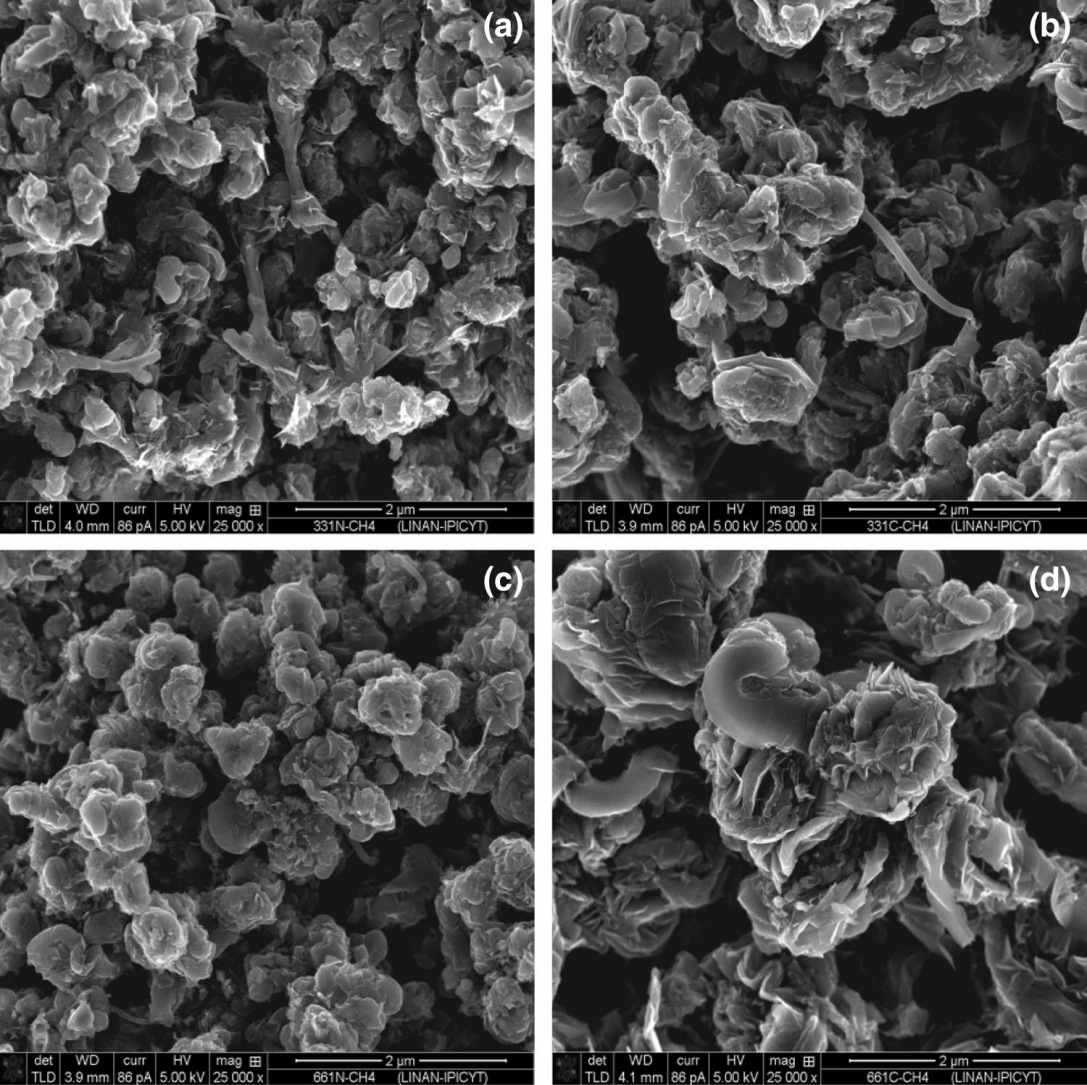
SEM post-reaction images of: **a** Co_3_Mo_3_N, **b** Co_3_Mo_3_C, **c** Co_6_Mo_6_N and **d** Co_6_Mo_6_C

**Fig. 7:**
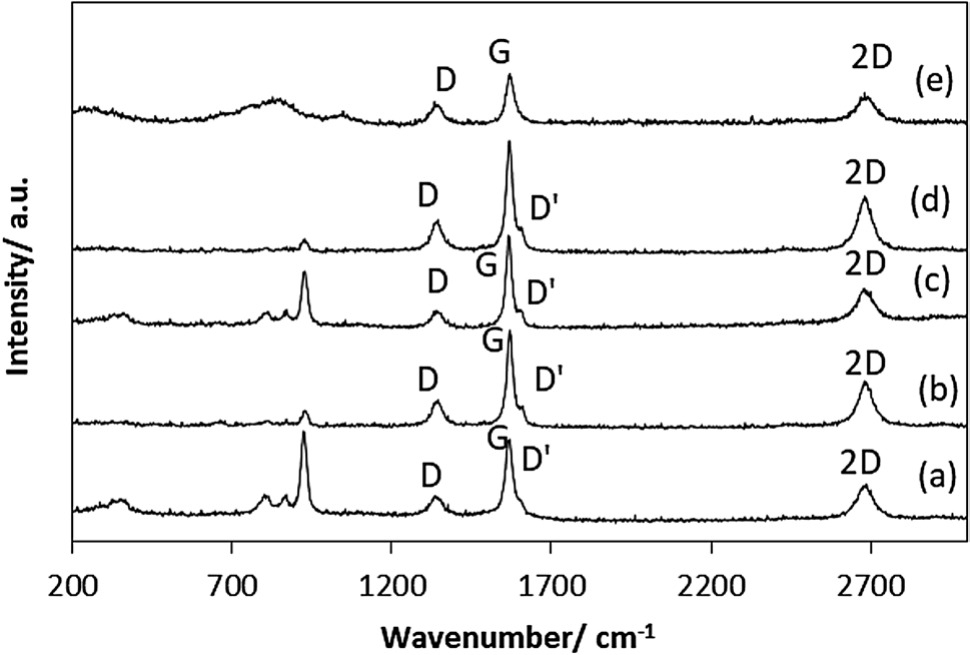
Raman spectra of the post-reaction materials (*a*) CoMoO_4_, (*b*) Co_3_Mo_3_N, (*c*) Co_3_Mo_3_C, (*d*) Co_6_Mo_6_N and (*e*) Co_6_Mo_6_C

**Fig. 8:**
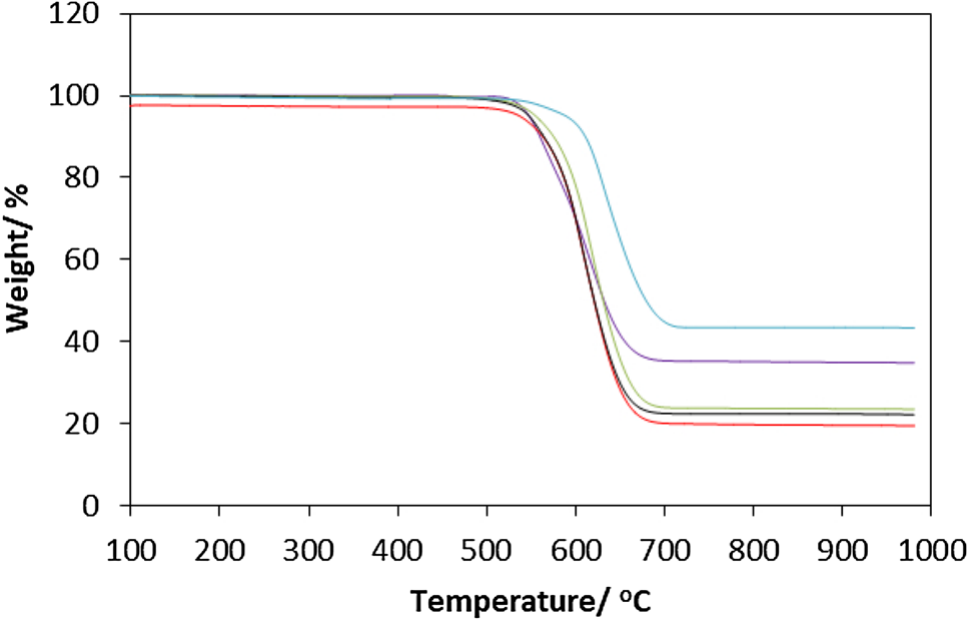
TGA traces under air for post-reaction materials: (purple) CoMoO_4_, (red) Co_3_Mo_3_N, (green) Co_3_Mo_3_C, (black) Co_6_Mo_6_N and (blue) Co_6_Mo_6_C

**Fig. 9:**
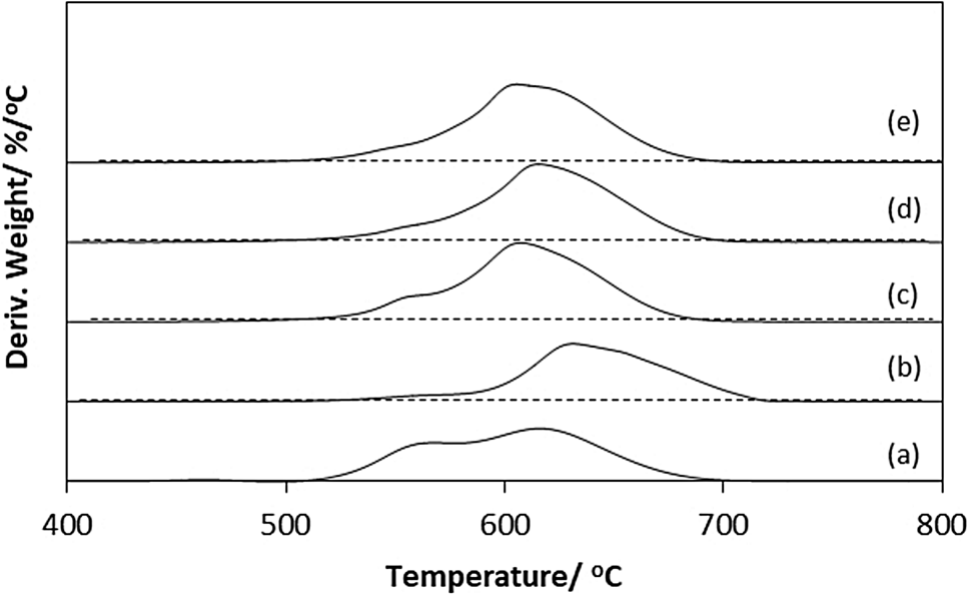
Derivative weight curves for post-reaction materials under air (*a*) CoMoO_4_, (*b*) Co_3_Mo_3_N, (*c*) Co_3_Mo_3_C, (*d*) Co_6_Mo_6_N and (*e*) Co_6_Mo_6_C

The characterisation of the post-reaction materials is presented in Figs. 5, 6, 7, 8 and 9 and in Table 1. Figure 5 presents the post-reaction powder XRD patterns. The XRD results are consistent with the formation of graphite as expected. In addition, a number of significant phase transformations to the original materials have occurred upon reaction (Fig. 5; Table 1). In most cases, Co_3_Mo_3_C appeared to be the predominant phase, although the formation of some β-Mo_2_C and α-Co is also apparent. However, interestingly, in the case of CoMoO_4_, only β-Mo_2_C, α-Co and graphite were evident after reaction with the ternary carbide phase being absent.

As shown in Fig. 6, morphological changes also occurred upon reaction. The agglomerated needle-like morphology was transformed to agglomerated block like structures. In contrast, post-reaction Co_6_Mo_6_C appeared to exhibit larger particle sizes compared to its post-reaction Co_3_Mo_3_N, Co_3_Mo_3_C and Co_6_Mo_6_N counterparts within the statistical limitations of the observations made. It is also interesting to note that in general, post-reaction catalyst displayed a higher surface area than pre-reaction materials. Both the drastic change in morphology and the enhanced surface area can be, at least partly, attributed to the significant deposition of carbon as a result of reaction. The comparatively lower surface area evident in the case of Co_6_Mo_6_C is in accordance with the large particle size observed for this sample by SEM.

The CoMoO_4_ and Co_6_Mo_6_C post-reaction samples were found to contain ~ 70 wt% C (Table 1), with a higher post-reaction carbon content (~ 84 wt% C) evident for Co_3_Mo_3_C, Co_3_Mo_3_N and Co_6_Mo_6_N as would be anticipated from their higher hydrogen formation rates. The nature of the carbonaceous species deposited was characterized by Raman spectroscopy. Post-reaction Raman spectra, presented in Fig. 7, provide strong evidence of the presence of disordered and graphitic carbon with Raman bands observed at 1350 and 1582 cm^−1^ labelled (D) and (G) respectively [25]. An additional band, of lower intensity, at 1620 cm^−1^ labelled as D′ was observed for the Co_3_Mo_3_N, Co_3_Mo_3_C and Co_6_Mo_6_N samples. In the literature, several explanations exist and the additional band can be correlated in principle to the presence of a high concentration of defects [26–28]. Additional Raman bands related to cobalt molybdate were detected in the post-reaction CoMoO_4_ and post-reaction Co_3_Mo_3_C.

To further characterise the nature of the carbon deposit after reaction, TGA in air was carried out over the temperature range 100–1000 °C (Fig. 8). It was observed that the carbon oxidation started for all materials from 500 °C and was complete by 700 °C. Beyond, this point no further change in the weight of the material was observed. The total weight loss was between 60 and 80 wt% which accords well with the results of post-reaction elemental analysis (Table 1). The first derivative profiles for the different post-reaction materials are presented in Fig. 9. In all materials, two weight loss regions have been observed. The first weight loss peak is observed in the temperature range between 500 and 600 °C while the second weight loss peak is observed at higher temperatures (600–700 °C). The contribution of the first peak was particularly significant in the post-reaction CoMoO_4_ material and to some extent in the Co_3_Mo_3_C. However, the oxidation of carbon in Co_3_Mo_3_N, Co_6_Mo_6_N and Co_6_Mo_6_C materials occurred mainly at temperature ranging between 600 and 700 °C. The contrast between the two oxidation regions could suggest the presence of two forms of carbon. However, the presence of the oxide phase, as evidenced in their Raman spectra, in the case of post-reaction CoMoO_4_ and post-reaction Co_3_Mo_3_C as observed by Raman spectra (Fig. 7) may have enhanced the oxidation of carbon at low temperature.

## Discussion

The topotactic transformation pathways and pseudomorphic nature of the cobalt molybdenum carbide and nitride families evident in this study offers, in principle, an elegant route to study the effect of interstitial carbon/nitrogen on the catalytic activity of cobalt molybdenum materials for methane cracking. All the prepared materials possess activity for hydrogen production at 800 °C. Interestingly, the catalytic activity of these materials varied depending upon initial composition with Co_6_Mo_6_N being the most active (Fig. 3). The activity of the material was found to stabilise around 1.8 mmol H_2_ g_catalyst_^−1^ min^−1^, which is high when compared against the activity of some other nitride systems (e.g. 180 µmol H_2_ g_catalyst_^−1^ min reported for a silicon-vanadium nitride nanocomposite under directly comparable conditions) [29]. In fact, the activity of the Co_6_Mo_6_N is directly comparable to the activity of iron oxide (for which a rate of 1 mmol H_2_ g_catalyst_^−1^ min^−1^ was previously reported for iron oxide under the same reaction conditions) [30]. In general, the activity of the carbides and nitrides presented a normalised hydrogen production rate ranging from 1.1 to 1.8 mmol H_2_ g_catalyst_^−1^ min^−1^. A slightly lower activity (0.8 mmol H_2_ g_catalyst_^−1^ min^−1^) was found for the CoMoO_4_ system. In addition to the relatively enhanced activity of the cobalt molybdenum carbide and nitride systems when compared to the oxide counterpart, the absence of significant production of CO_x_, beyond that which might be expected from conversion of the surface passivation layer, during the methane cracking reaction is of potential interest in relation to free of CO_x_-H_2_ production.

As expected, the production of H_2_ was accompanied by carbon deposition. Due to the nature of the reaction, the amount of carbon deposited on carbide and nitride systems can be correlated directly to the activity of methane cracking as expected. Elemental analysis showed significant deposition of carbon ~ 85 wt% on Co_3_Mo_3_N, Co_3_Mo_3_C and Co_6_Mo_6_N confirming the high activity of these materials, in spite of the less reproducible hydrogen production behaviour of Co_3_Mo_3_C. Thermogravimetric analyses conducted under air confirmed that the weight loss associated with carbon oxidation to be consistent with the elemental analyses of post-reaction materials. The nature of the carbon present upon reaction has been investigated by Raman spectroscopy (Fig. 7). The Raman futures were dominated by the presence of two forms of carbon: disordered and graphitic carbon. The existence of several forms of carbon was also evident from the derivative weight curves for post-reaction samples.

While it is tempting to discuss the activity of the catalysts against their initial composition, post-reaction analysis revealed changes in the structural properties upon reaction. As might be expected under the reaction conditions applied, post-reaction powder XRD (Fig. 5), showed the carburisation of all the materials studied when reacted. However, the products of carburisation slightly differ depending on the initial composition. In the post-reaction CoMoO_4_, only β-Mo_2_C (001-1188) and α-Co (01-089-7093) are observed as a result of the carburisation of CoMoO_4_ as well as graphite generated from methane cracking. However, a mixture of Co_3_Mo_3_C (03-065-7128), α-Co (01-089-7093) and β-Mo_2_C (001-1188) is detected upon reaction of Co_3_Mo_3_N, Co_3_Mo_3_C and Co_6_Mo_6_N with methane. In the case of Co_6_Mo_6_C relocation of the carbon located in the 8a (0 0 0) Wyckoff site to the 16c (1/8 1/8 1/8) site occurs associated with the formation of Co_3_Mo_3_C without clear evidence of the formation of β-Mo_2_C and α-Co [01-089-7093 (001-1188)] phases as observed in the previous cases.

In summary, for the three most active catalysts, the phases detected after reaction comprised a mixture of Co_3_Mo_3_C (03-065-7128), α-Co (01-089-7093) and β-Mo_2_C (001-1188). While, for the least active material CoMoO_4_, only the β-Mo_2_C and α-Co (01-089-7093) were detected. Another major aspect, where differences are potentially evident, is the accessible surface area of the active phases. The surface area measured in post-reaction Co_3_Mo_3_N, Co_3_Mo_3_C and Co_6_Mo_6_N samples ranged between 50 and 63 m^2^ g^−1^ while in the case of CoMoO_4_ and Co_6_Mo_6_C the surface area was limited to ~ 30 m^2^ g^−1^. Despite the fact that no simple link can be established between the catalytic activity to phase composition and accessible surface area, it can be argued that the presence of Co_3_Mo_3_C, α-Co and β-Mo_2_C and high surface area leads to an enhanced activity for methane cracking. In addition, the initial composition seems to play an important role in the final activity of the catalysts. These differences may indicate differences in the active surface composition resulting from the carburisation process of different cobalt molybdenum precursors. Further characterisation of the carburisation process of cobalt molybdenum materials by in-situ neutron diffraction, in condition of relevance to this study, are currently under investigation and will bring new insight to the process. Elsewhere, cobalt-molybdenum oxycarbide surface phases have been proposed to be of importance for activity and lifetime [31]. 

## Conclusion

A range of cobalt molybdenum containg materials have been prepared and tested as catalysts for the production of hydrogen from methane at 800 °C. After a short induction period, all samples were active and stable for the generation of hydrogen over the period tested, with the exception of Co_3_Mo_3_C. Amongst the evaluated materials, the Co_6_Mo_6_N sample showed the highest activity of about 1.8 mmol H_2_ g^−1^ min^−1^, comparable to those observed for iron oxide systems under similar reaction conditions. The results revealed that a significant phase transformation from metal nitrides to Co_3_Mo_3_C and β-Mo_2_C occurred throughout the methane cracking reaction. Interestingly, in the case of Co_6_Mo_6_C relocation of the carbon located in the 0 0 0 (8a) site to 1/8 1/8 1/8 (16c) sites resulting in the formation of Co_3_Mo_3_C was observed. Furthermore, results from Raman spectroscopy and powder XRD show that at least two forms of carbon are formed on the catalyst surface during methane decomposition.

## Electronic supplementary material

Below is the link to the electronic supplementary material.

Supplementary material 1 (DOCX 6673 KB)

Supplementary material 1 (DOCX 6673 KB)
