# Supplementary material for: Methane Cracking over Cobalt Molybdenum Carbides
Source: Catal Letters. 2018 Apr 21;148(6):1643–50. doi: 10.1007/s10562-018-2378-4 (PMC6448361; doi:10.1007/s10562-018-2378-4)
Supplement: Supplementary file 1 — Supplementary material 1 (DOCX 6673 KB) [file 10562_2018_2378_MOESM1_ESM.docx]

Supplementary information

**Methane cracking over cobalt molybdenum carbides**

**I. Alshibane^a^, S. Laassiri^a^, J. L. Rico^b^, J. S. J. Hargreaves*^a^**

^a^ WestCHEM, School of Chemistry, Joseph Black Building, University of Glasgow, Glasgow G12 8QQ, UK.

^b^ Laboratorio de Catálisis, Facultad de Ingeniería Química, Universidad Michoacana de San Nicolás de Hidalgo, Edif. V1, C.U., CP 58060, Morelia, Mich., México.

E-mail: [I.alshibane.1@research.gla.ac.uk](mailto:I.alshibane.1@research.gla.ac.uk), ORCID: https://orcid.org/0000-0001-6410-3680

E-mail: Said.Laassiri@glasgow.ac.uk, ORCID ID: https://orcid.org/0000-0003-4075-5806

E-mail: Jlceri@yahoo.com.mx

*E-mail: Justin.Hargreaves@glasgow.ac.uk, ORCID ID: https://orcid.org/0000-0003-1926-9299

**Figure S1**. Powder X-ray diffraction patterns of (a) CoMoO_4_, (b) Co_3_Mo_3_N, (c) Co_3_Mo_3_C, (d) Co_6_Mo_6_C and (e) Co_6_Mo_6_N.


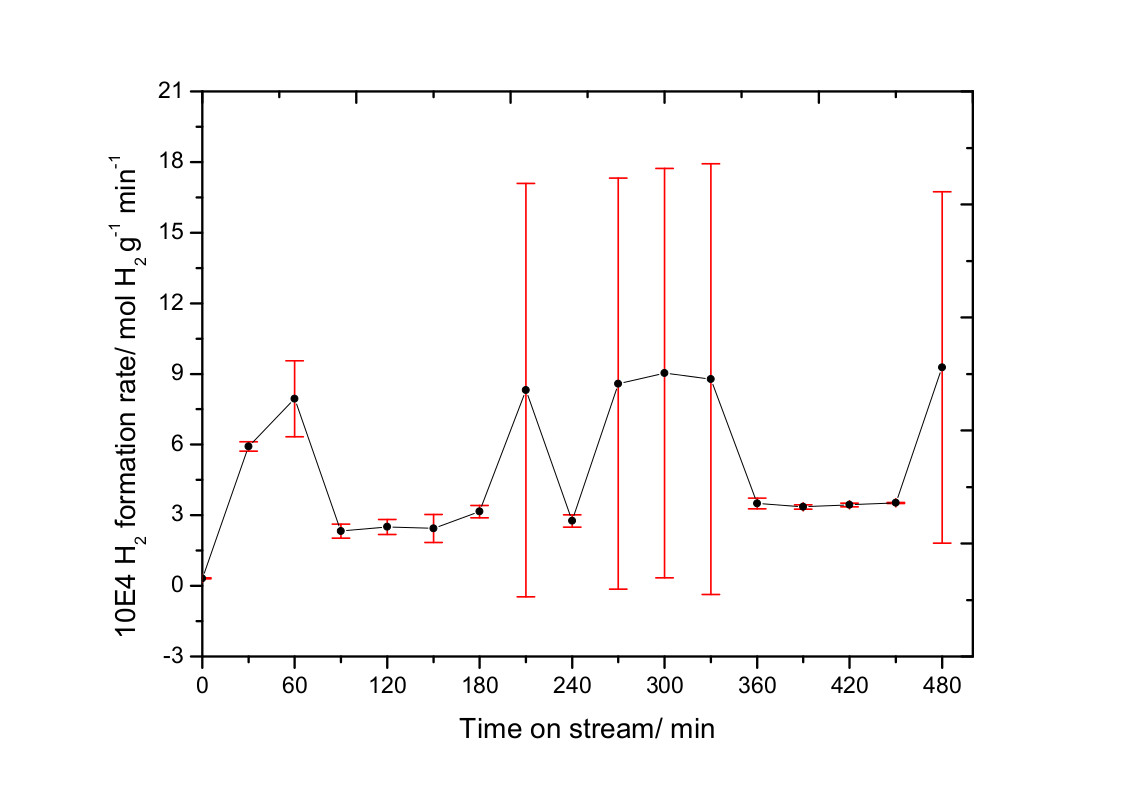


**Figure S2**. Repeatability study of hydrogen formation rate as a function of time on stream for CH_4_ cracking over Co_3_Mo_3_C material at 800 °C.

**Figure S3.** PXRD patterns of replicate post-reaction Co_3_Mo_3_C. (a) first sample and (b) second sample

**Table S.1.** Summary of the textural and structural characterisation of post-reaction Co_3_Mo_3_C replicates

|  | Post-reaction XRD Phase | Carbon content / wt.% | | S_BET_/ m^2^.g^-1^ | |
| --- | --- | --- | --- | --- | --- |
|  |  | as-prepared | post-reaction | as prepared | post-reaction |
| Co_3_Mo_3_C | Graphite (003-0401), β-Mo_2_C (001-1188), α-Co (01-089-7093) | 2.5 | 79 | 14 | 51 |
| Co_3_Mo_3_C | Graphite (003-0401), β-Mo_2_C (001-1188), α-Co (01-089-7093) | 2.5 | 84 | 13 | 50 |

**Figure S4.** TGA s of post-reaction Co_3_Mo_3_C samples under air from RT to 1000 °C.

**Figure S5.** First derivatives of post-reaction Co_3_Mo_3_C samples under air from RT to 1000 °C.

(a)

| 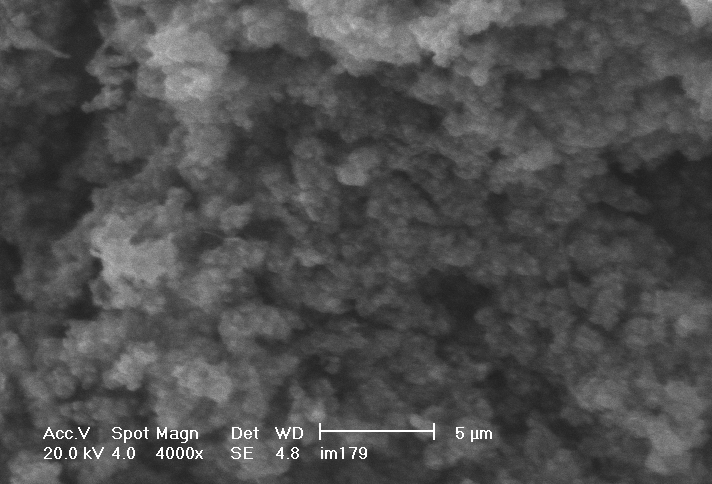 | **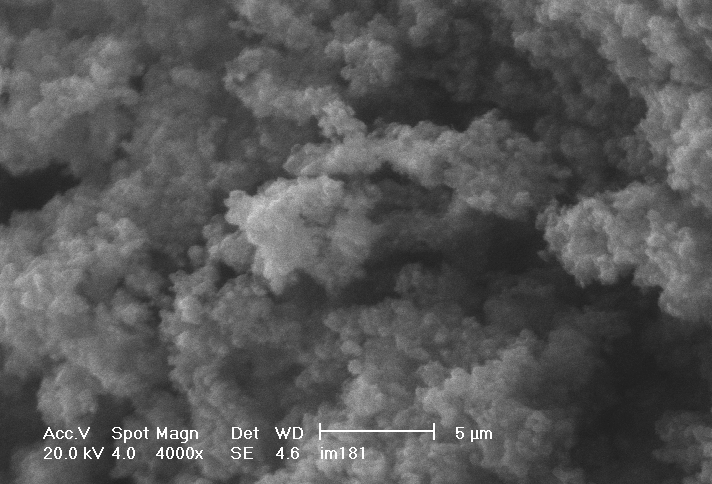**  (b) |
| --- | --- |

**Figure S6.** SEM images of post-reaction replicate Co_3_Mo_3_C samples. (a) first sample (b) second sample.

**Figure S7.** Raman spectra of replicate post-reaction Co_3_Mo_3_C samples. (a) first sample, (b) second sample
